# Supplementary material for: In vitro assembly, positioning and contraction of a division ring in minimal cells
Source: Nat Commun. 2022 Oct 15;13:6098. doi: 10.1038/s41467-022-33679-x (PMC9569390; doi:10.1038/s41467-022-33679-x)
Supplement: Supplementary file 1 — Supplementary Information [file 41467_2022_33679_MOESM1_ESM.pdf]

Supplementary Information

In vitro assembly, positioning and contraction of a division ring in minimal cells

Shunshi Kohyama[1,§], Adrián Merino-Salomón[1,§] and Petra Schwille[1,*]

[§]These authors contributed equally

[1]Dept. Cellular and Molecular Biophysics, Max Planck Institute of Biochemistry, Am Klopferspitz 18, 82152 Martinsried, Germany. E-mail: schwille@biochem.mpg.de

Supplementary Figures

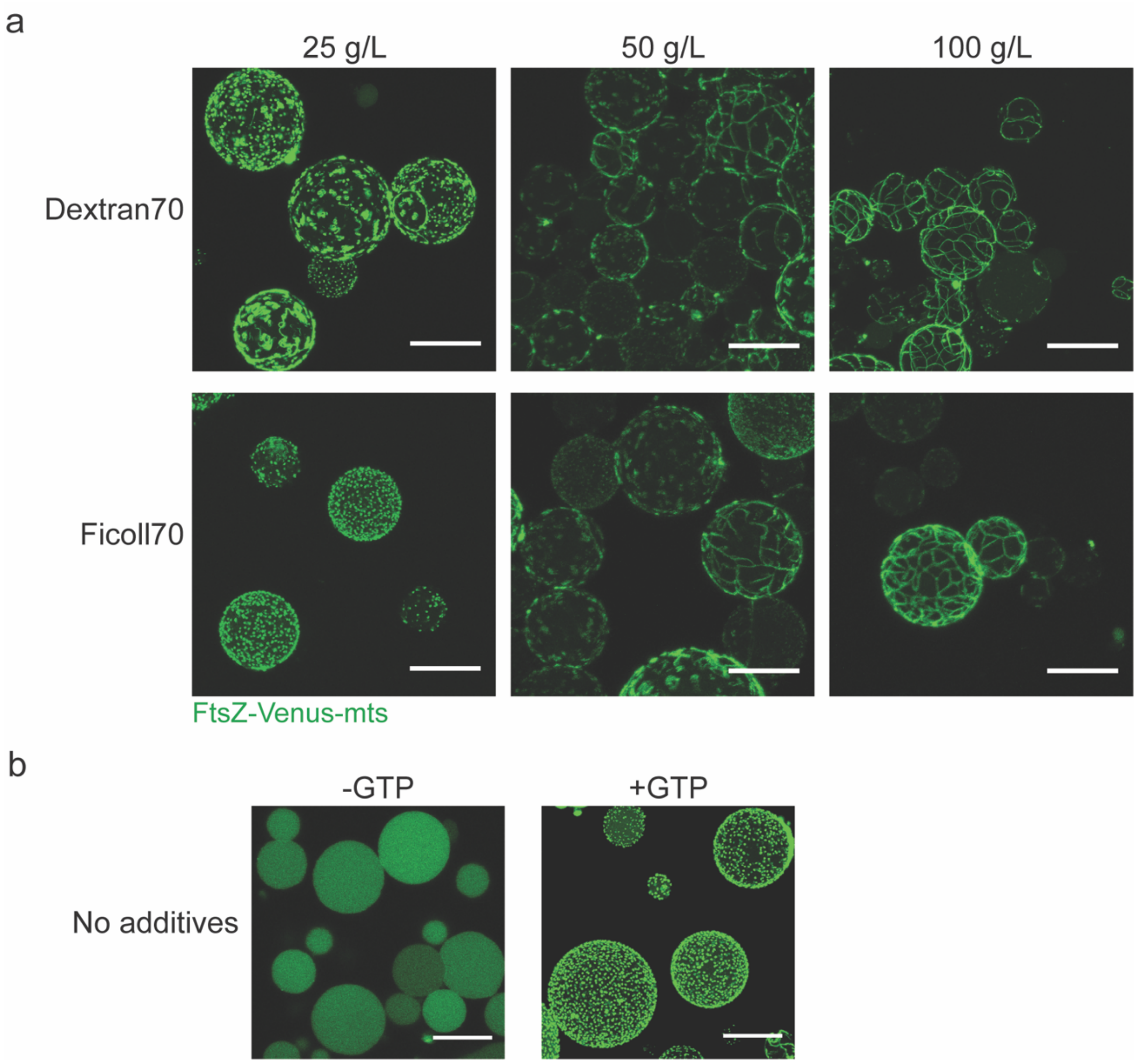

Supplementary Fig. 1 Effect of macromolecular crowding over FtsZ behavior inside lipid vesicles. **a** Representative 3D max projection images of FtsZ-Venus-mts structures inside lipid vesicles under different crowding conditions using Dextran70 and Ficoll70 (2 μM FtsZ-Venus-mts + 1 mM GTP). Scale bars: 25 μm. **b** Representative 3D max projection images of FtsZ-Venus-mts (2 μM) without the addition of crowders with the absence (above) and presence (below) of 1 mM GTP. Scale bars: 25 μm.

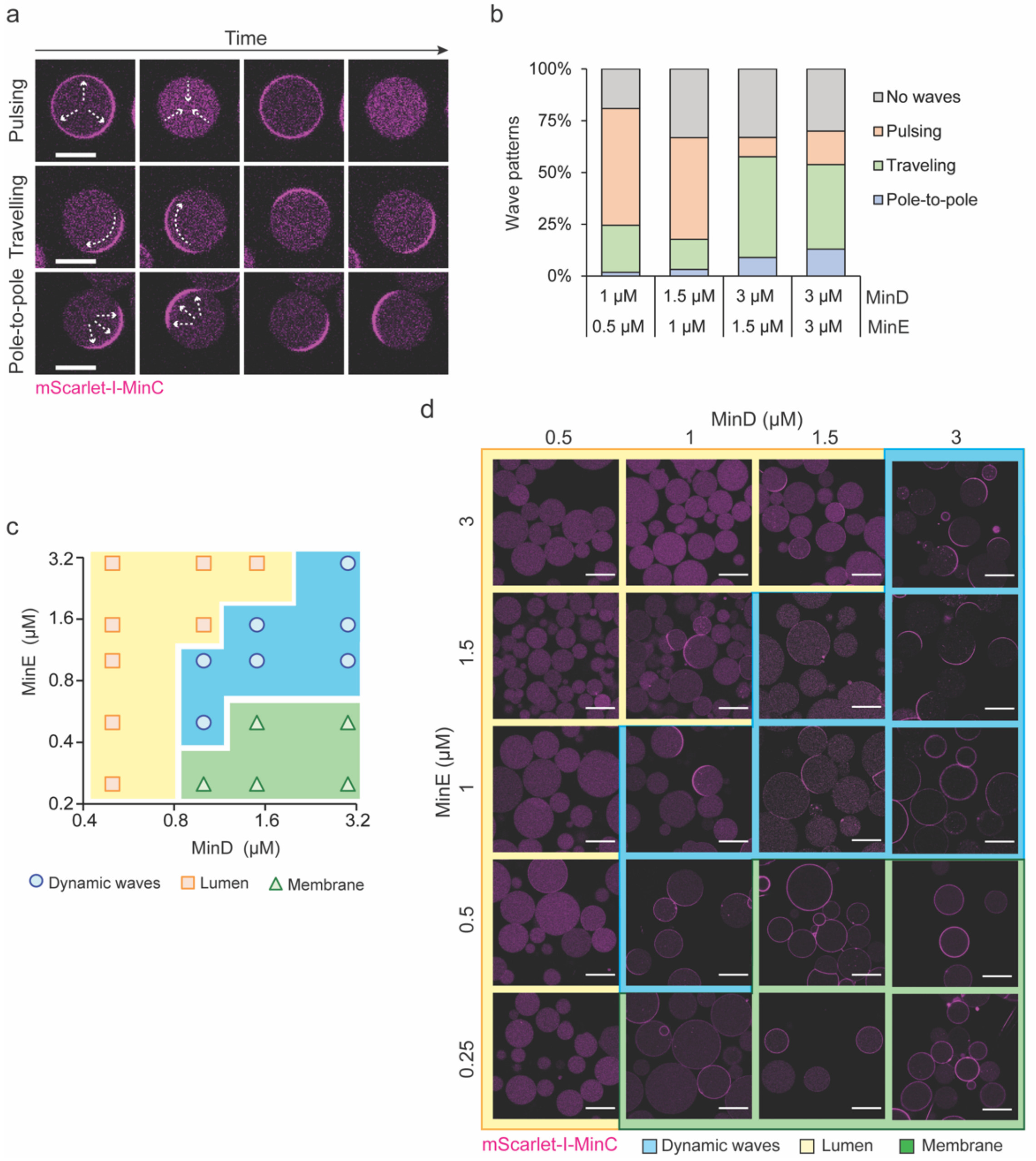

Supplementary Fig. 2 Reconstitution of the MinCDE system inside lipid vesicles. **a** Encapsulation of MinCDE proteins within lipid vesicles result in different Min wave patterns that can be categorized in three main groups depending on their behavior. Pulsing oscillations: MinD oscillates between the lumen of the vesicles and the membrane surface. Travelling or circling: Min proteins continuously revolve on the membrane of the GUV. Pole-to-pole oscillations: Min proteins bind alternately to the membrane of the two hemispheres of the vesicle. White arrows represent the behavior of MinCDE proteins inside vesicles. Scale bars: 15 μm. **b** Frequency of Min wave patterns at different MinDE ratios. Min wave patterns are differentiated into four

categories: absence of waves, pole-to-pole oscillations, traveling waves, or pulsing inside vesicles at different MinDE ratios (n = 220 for each condition). The four MinDE ratios that showed the relatively high percentage (>65%) of dynamic waves were selected for this analysis. **c** Phase diagram of major MinCDE pattern formation inside lipid vesicles without crowding conditions. To facilitate their interpretation, we have grouped the three MinCDE wave patterns (Pulsing, travelling or Pole-to-pole) into the category of "Dynamic" here in contrast with localization in lumen (No significant membrane attachment or protein waves), and localization on membrane (No MinCDE waves). These three categories are represented vs. MinDE concentrations. **d** Representative confocal images of lipid vesicles containing MinCDE proteins at different MinDE ratios. Scale bars: 25 µm. Source data are provided as a Source Data file.

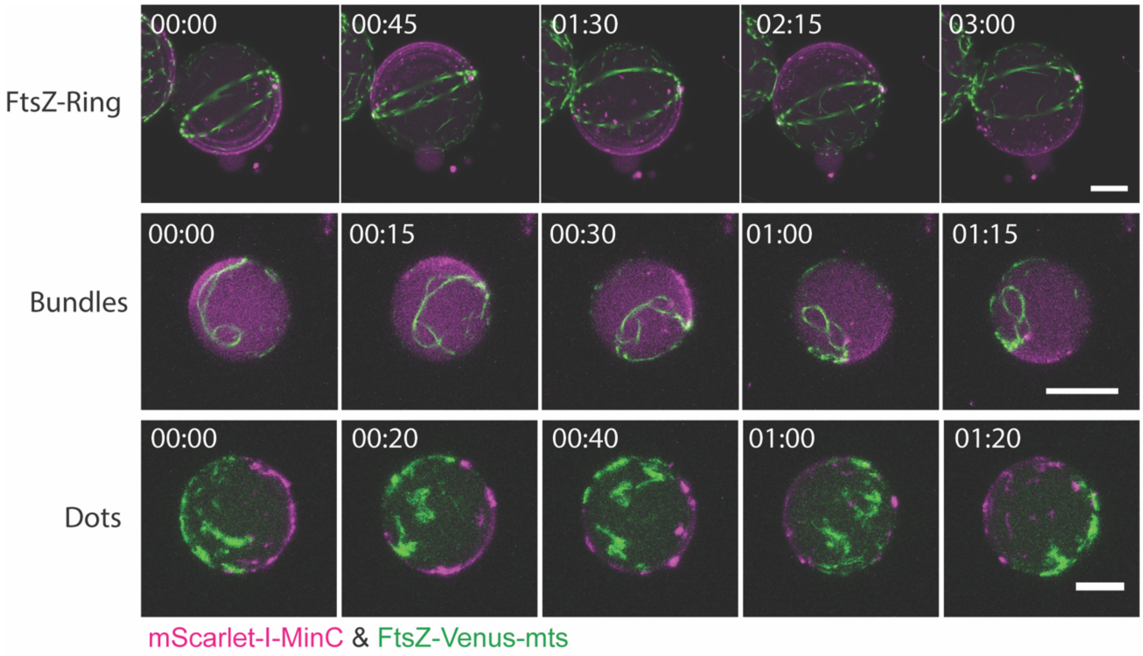

Supplementary Fig. 3 FtsZ structures are reorganized by MinCDE oscillations inside vesicles. Time-lapse confocal images of FtsZ-Venus-mts structures in the presence of MinCDE proteins. FtsZ structures bundles/mesh or dots are reorganized by the MinCDE waves, sometimes forming FtsZ-rings (2μM FtsZ-Venus-mts, 3μM MinD, 3μM MinE, 0.5μM mScarlet-I-MinC). Time is represented as mm:ss and Scale bars: 10 μm. Confocal images were recorded after 10 min of vesicle preparation for 1-2h.

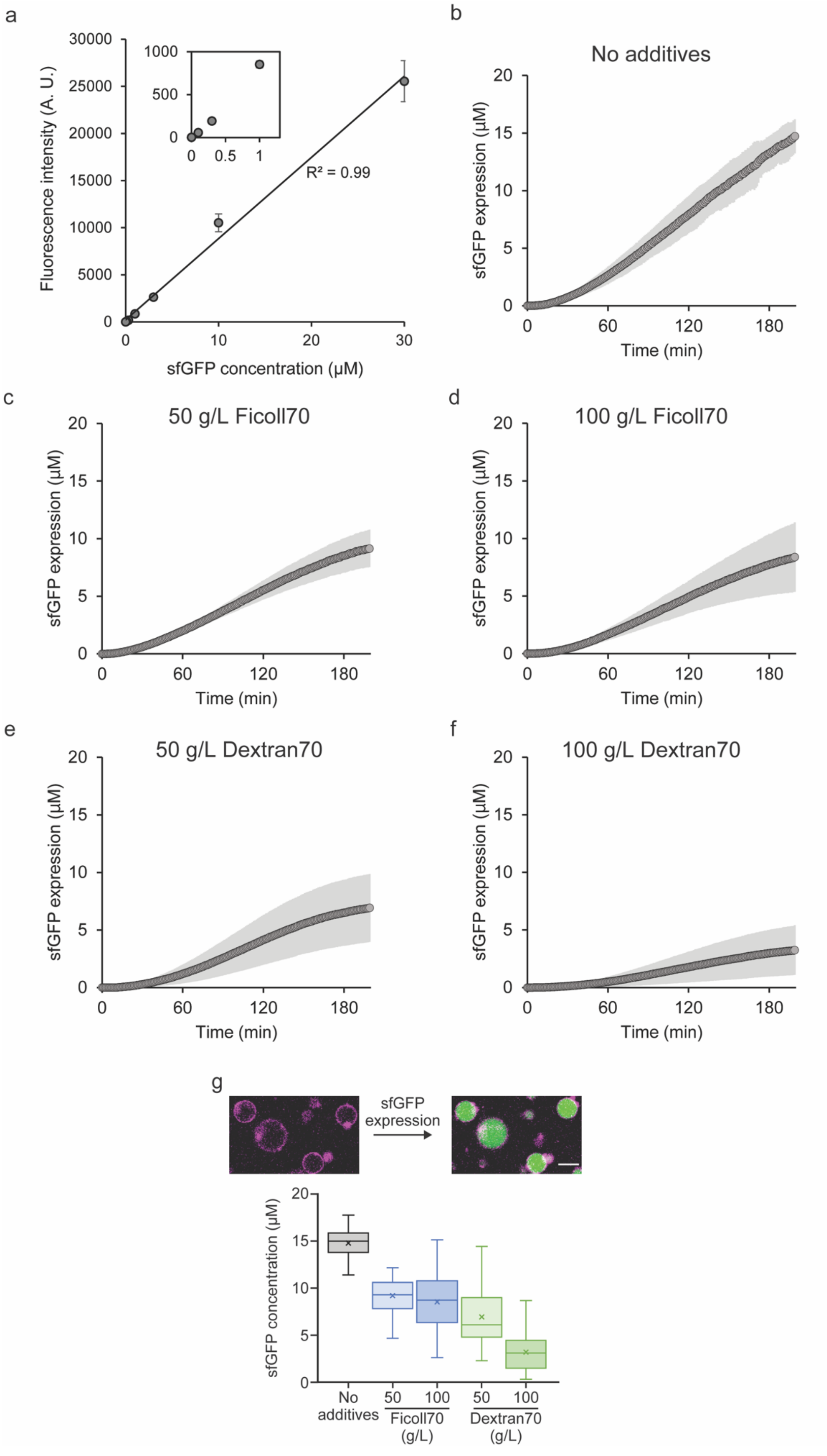
a
Fluorescence intensity (A. U.)
sfGFP concentration (μM)
R² = 0.99
b
No additives
c
50 g/L Ficoll70
d
100 g/L Ficoll70
e
50 g/L Dextran70
f
100 g/L Dextran70
sfGFP expression (μM)
Time (min)
g
sfGFP expression
sfGFP concentration (μM)
No additives
Ficoll70 (g/L)
Dextran70 (g/L)

Supplementary Fig. 4 Estimation of cell-free expression level under macromolecular crowding environment within lipid vesicles. **a** Standard curve of sfGFP concentration against fluorescence intensity (Arbitrary Units) within vesicles. 0, 0.1, 0.3, 1, 3, 10, and 30 μM of sfGFP were encapsulated within vesicles, and the mean fluorescence intensities of sfGFP were measured. Plots represent the mean intensity and standard deviation. n = 30 for each concentration. **b**-**f** Time-development of sfGFP expression under macromolecular crowding environments. sfGFP was expressed using 2 nM *sfGFP* template inside vesicles with no additives (**b**), 50 g/L Ficoll70 (**c**), 100 g/L Ficoll70 (**d**), 50 g/L Dextran70 (**e**), or 100 g/L Dextran70 (**f**). Then, 100 different vesicles were randomly chosen, and the fluorescence intensity of sfGFP in each vesicle was measured every 1 min for 200 min. Plots and bars indicate means and standard deviations, respectively. **g** Cell-free expression of sfGFP within vesicles under different crowding conditions. Top: Expression of sfGFP under 50 g/L of Ficoll7. Images show the same vesicles before incubation and after 200 min incubation at 37 °C. Scale bar: 20 μm. Bottom: Box plot of expression levels of sfGFP inside vesicles after 200 min incubation at 37 °C. Under Ficoll70 crowding, sfGFP expression level reached around 10 μM, while Dextran70 radically impaired expression. Mean is shown as a cross mark, box limits are quartiles 1 and 3, line inside boxes indicate median, and whiskers are the highest or lowest data points. n = 100 for each crowding condition. Source data are provided as a Source Data file.

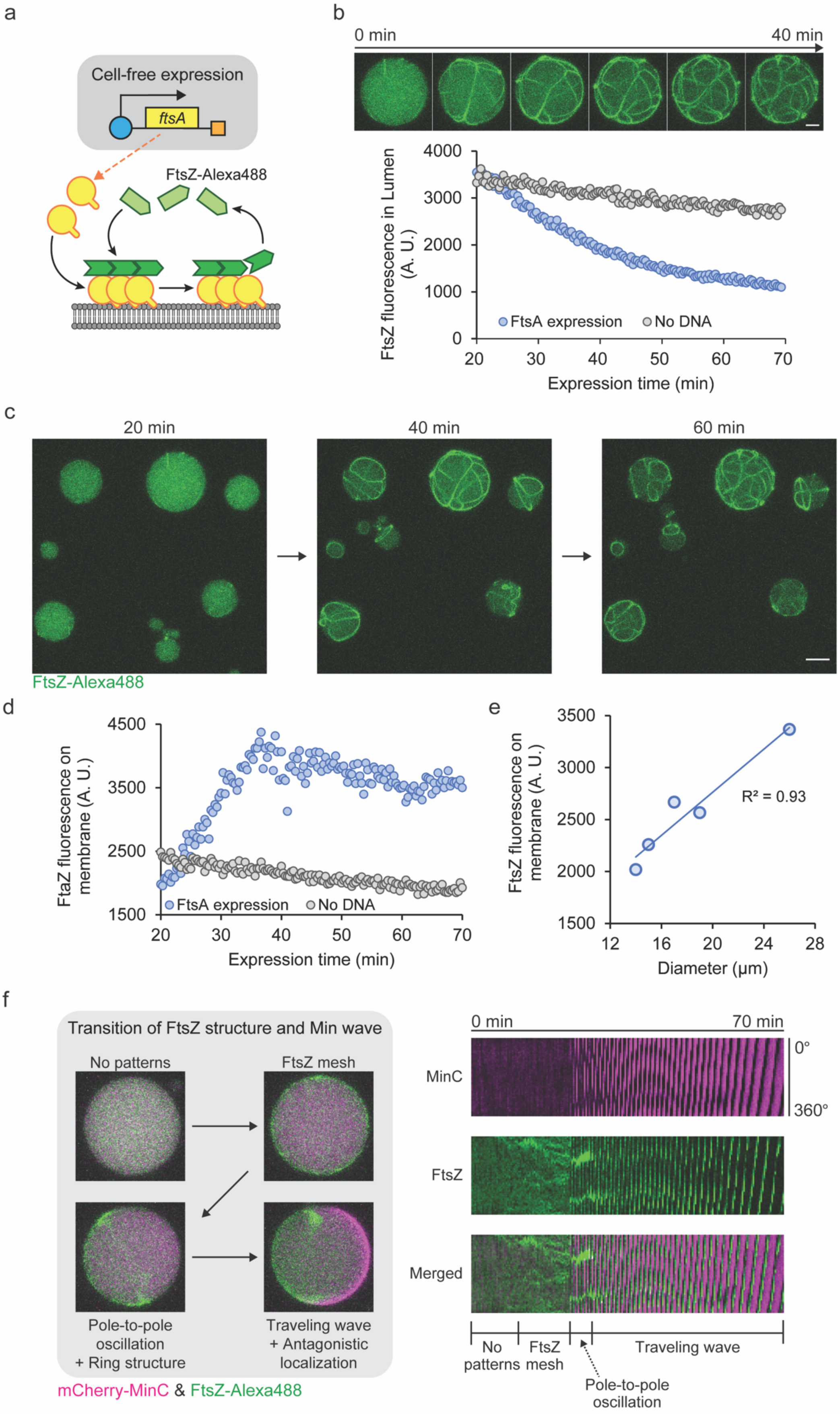
a
Cell-free expression
ftsA
FtsZ-Alexa488
b
0 min
40 min
FtsZ fluorescence in Lumen (A. U.)
FtsA expression
No DNA
Expression time (min)
c
20 min
40 min
60 min
FtsZ-Alexa488
d
FtaZ fluorescence on membrane (A. U.)
FtsA expression
No DNA
Expression time (min)
e
FtsZ fluorescence on membrane (A. U.)
R² = 0.93
Diameter (μm)
f
Transition of FtsZ structure and Min wave
No patterns
FtsZ mesh
Pole-to-pole oscillation + Ring structure
Traveling wave + Antagonistic localization
mCherry-MinC & FtsZ-Alexa488
0 min
70 min
MinC
FtsZ
Merged
0°
360°
No patterns
FtsZ mesh
Traveling wave
Pole-to-pole oscillation

Supplementary Fig. 5 Development of the FtsZ structures within lipid vesicles through cell-free expression of FtsA and MinDE proteins. **a** Schematic illustration of PURE cell-free expression of FtsA with purified FtsZ. **b** FtsZ-FtsA mesh structure inside vesicles via FtsA expression. Top: Time-lapse images of FtsZ mesh structure formation with 3D max projection. FtsZ indicates dynamic assembly into bundles on the membrane inside the vesicle along with FtsA expression using 5 nM of *ftsA* template with 2 µM purified FtsZ-Alexa488 and 50 g/L Ficoll70. Recording started after 20 min of cell-free expression. Scale bar: 5 µm. Bottom: localization change of FtsZ-Alexa488 along with FtsA expression in the lumen of the vesicles. FtsZ level (Arbitrary Units) decreases in the lumen by expressing FtsA **(**5 nM of *ftsA* template), targeting FtsZ to the membrane, while the negative control (no DNA) indicates a slight decrease of fluorescence by photobleaching. **c** Time-lapse images of FtsZ-FtsA mesh development with 3D max projection. FtsA was expressed at 37 °C using 5 nM of *ftsA* template with 2 µM purified FtsZ-Alexa488 and 50 g/L Ficoll70. FtsZ indicated dynamic assembly into mesh structures on the membrane along with time-lapse. Scale bar: 20 µm. **d** Localization change of FtsZ-Alexa488 along with FtsA expression on membrane of the vesicles. In contrast to the FtsZ intensity in the lumen (Supplementary Fig. 5b), FtsZ level (Arbitrary Units) increased by expressing FtsA, reaching its maxima between 30-40 min of expression and later slightly decreased the fluorescence probably due to the photobleaching. **e** Size dependency of the FtsZ intensity on lipid membrane among vesicles. After 80 min of cell-free expression of FtsA (with 5 nM of *ftsA* template), fluorescent intensity of FtsZ-Alexa488 (Arbitrary Units) tended to be higher within larger vesicles, suggesting that concentration of FtsZ on membrane become higher in larger vesicles. **f** Regulation of FtsZ localization by stable Min traveling waves via co-expression of FtsA, MinD, and MinE (with 2 nM *ftsAopt* and 2 nM *minDEoperon*). Left: 3D max projections of the transition of Min wave patterns and corresponding FtsZ structures. Right: Kymographs of the pattern transition of Min waves and FtsZ structures. Compared to Fig. 3c, pole-to-pole oscillations of Min waves were unstable and steady at traveling waves. Recording started after 5 min of cell-free expression. mCherry-MinC and FtsZ-Alexa488 are indicated in magenta and green, respectively. Source data are provided as a Source Data file.

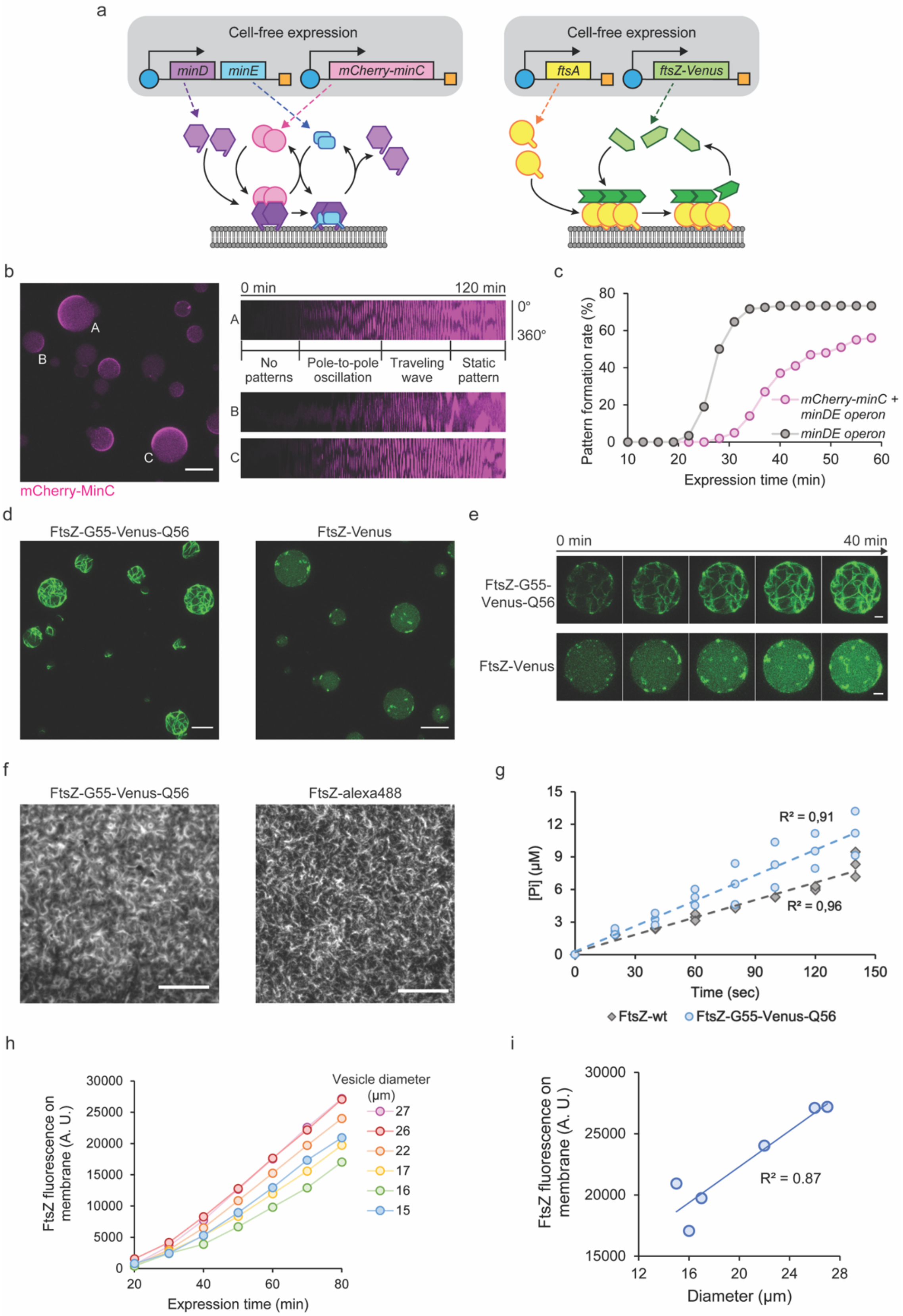

Supplementary Fig. 6 Independent reconstitution of MinCDE and FtsZ-FtsA systems

by co-expressing all essential components. **a** Schematic illustrations of co-expression systems. Left: Co-expression of mCherry-MinC, MinD, and MinE. Right: Co-expression of FtsA and FtsZ-Venus mutant. **b** Emergence of Min waves by cell-free expressed mCherry-MinC, MinD, and MinE inside vesicles (with 2 nM *mCherry-MinC* and 1 nM *minDEoperon*). Left: 3D max projection of Min wave pattern formation inside vesicles after 2 h expression of mCherry-MinC, MinD, and MinE at 37 °C. Right: Kymographs of the Min waves inside vesicles correspond to the same letters in the left image captured by mCherry-MinC fluorescence. Wave dynamics were highly sensitive to MinDE concentrations. Scale bar: 20 µm. **c** Time-dependent occurrence of Min wave patterns inside lipid vesicles. Percentage of Min patterns (dynamic waves and static patterns) among vesicles are calculated every 3 min after 10 min incubation. 0.5 nM *minDEoperon* (black points and gray lines) or 1 nM *mCherry-MinC* + 0.5 nM *minDEoperon* (magenta points and pink lines) were used for MinDE or MinCDE co-expression, respectively. n = 116 and 100 for MinDE and MinCDE expression, respectively. **d** 3D max projection of the FtsZ-FtsA mesh structures inside vesicles after 1 h co-expression of either FtsZ-G55-Venus-Q56 or FtsZ-Venus (3 nM *ftsZopt-G55-Venus-Q56* or *ftsZ-Venus*) together with FtsA (1 nM *ftsAopt*) at 37 °C. In contrast to FtsZ-G55-Venus-Q56, FtsZ-Venus mostly localized in the lumen of vesicles and formed several dot structures on the membrane. Scale bars: 20 µm. **e** Comparison of FtsZ mesh formation dynamics for FtsZ-G55-Venus-Q56 and FtsZ-Venus. FtsZ-G55-Venus-Q56 efficiently forms mesh structures on the membrane via co-expression with FtsA, while FtsZ-Venus forms dot-like structures. Experimental conditions were the same as Supplementary Fig. 4d and images were recorded after 20 min incubation at 37 °C. Scale bars: 5 µm. **f** Purified FtsZ bundles on SLBs through FtsA. FtsZ-G55-Venus-Q56 (left) and wild type FtsZ (right) were anchored to the membrane through FtsA and assembled into smilar dynamic vortices. Scale bars: 10 µm. **g** Release of Pi as result of GTPase activity of FtsZ-G55-Venus-Q56 and wild type FtsZ. FtsZ-G55-Venus-Q56 has slightly higher GTPase activity (~6.2 Pi/FtsZ * min^{-1}) than wild type FtsZ (~4.2 Pi/FtsZ * min^{-1}), thus, both proteins are active under our experimental conditions (n=3). Plots represent the mean value of Pi release at each time point. GTPase activity of FtsZ proteins was determined from the slope of the linear phosphate release. **h** FtsZ-G55-Venus-Q56 intensity on the vesicle membrane along with co-expression of FtsA and FtsZ-G55-Venus-Q56 (3 nM *ftsZopt-G55-Venus-Q56* and 1 nM *ftsAopt*) at 37 °C. Similar to the Supplementary Fig. 5b, intensity of the FtsZ (Arbitrary Units) gradually increased with time by binding of FtsZ-G55-Venus-Q56 on the membrane through FtsA. Although similar trends were observed among different vesicle sizes, fluorescence intensities tended to be slightly higher within larger vesicles. **i** Size

dependency of the FtsZ-G55-Venus-Q56 intensity on lipid membrane among vesicles. After 80 min of co-expression of FtsA and FtsZ-G55-Venus-Q56 (with 3 nM *ftsZopt-G55-Venus-Q56* and 1 nM *ftsAopt*), fluorescent intensity of FtsZ (Arbitrary Units) indicated the correlation between vesicle diameter and FtsZ concentration on membrane. Source data are provided as a Source Data file.

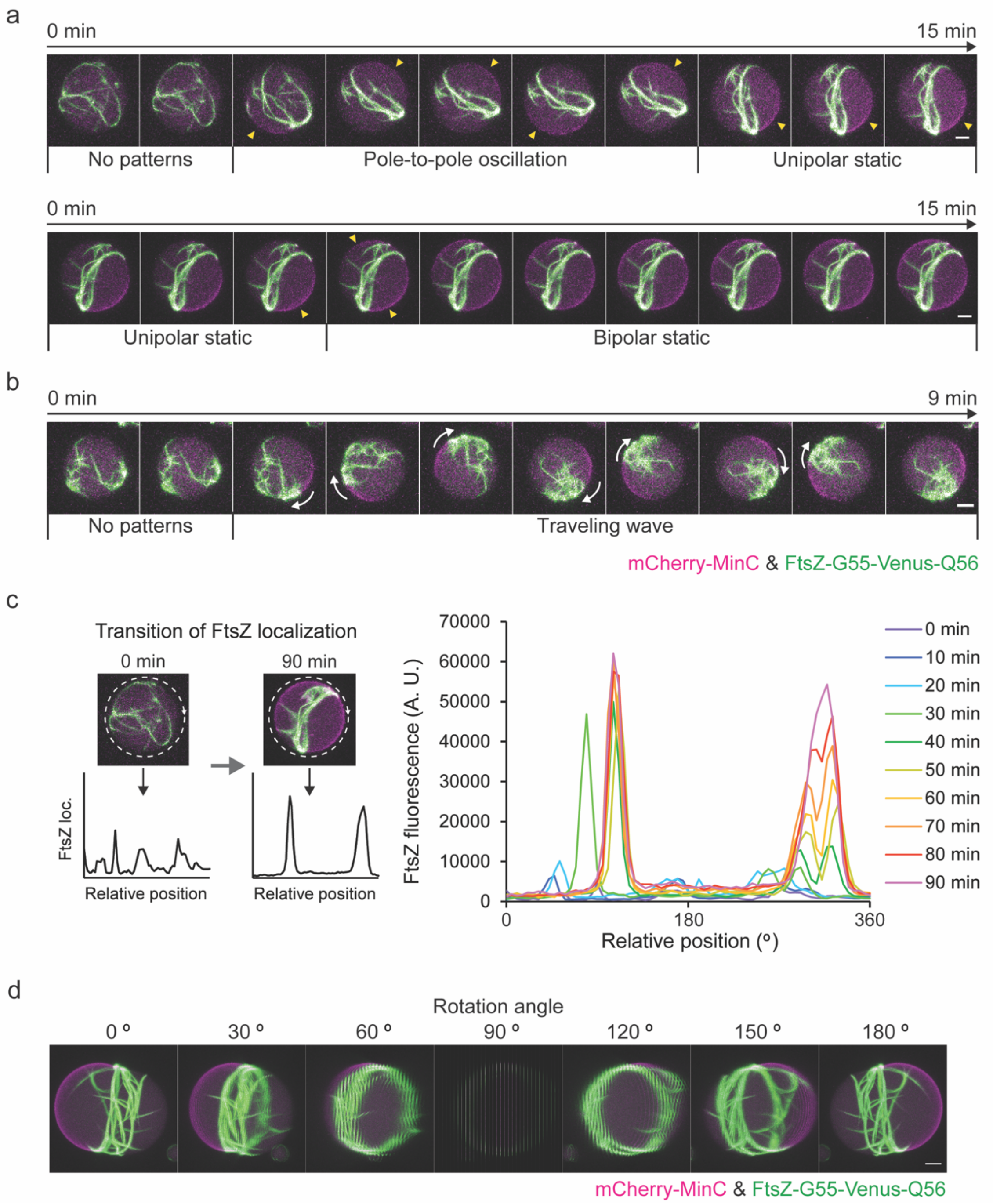

Supplementary Fig. 7 FtsZ-ring structures regulated by various Min wave patterns inside lipid vesicles in co-expression system (1 nM *ftsAopt*, 3 nM *ftsZopt-G55-Venus-Q56*, 2 nM *mCherry-MinC*, and 1 nM *minDEoperon* templates). **a** Time-lapse images of the formation of the FtsZ-ring like structure regulated by the pole-to-pole oscillation of Min waves. The static localization of Min waves further stabilized the FtsZ structure. Scale bars: 5 μm. **b** Time-lapse images of the moving of FtsZ mesh structure driven by Min traveling waves around the membrane. Scale bar: 5 μm. **c** Surface plot of the FtsZ

distribution regulated by Min waves within a vesicle. Fluorescence intensities of FtsZ-G55-Venus-Q56 (Arbitrary Units) on the equatorial plane of the vesicle were measured along with time. The FtsZ was randomly distributed initially but then formed two peaks that indicate the formation of the FtsZ structure on the membrane by regulation of Min waves. **d** 3D rotational view of a constricted vesicle by the FtsZ-ring. 3D max projected image was rotated along with Y-axis in 30 ° increment between each frame and 7 (0 ° – 180 °) frames were shown. The image was captured started after 3 h of incubation at 37 °C. Scale bar: 5 μm. All images are 3D max projection with the merged color of mCherry-MinC (magenta) and FtsZ-G55-Venus-Q56 (green). Source data are provided as a Source Data file.

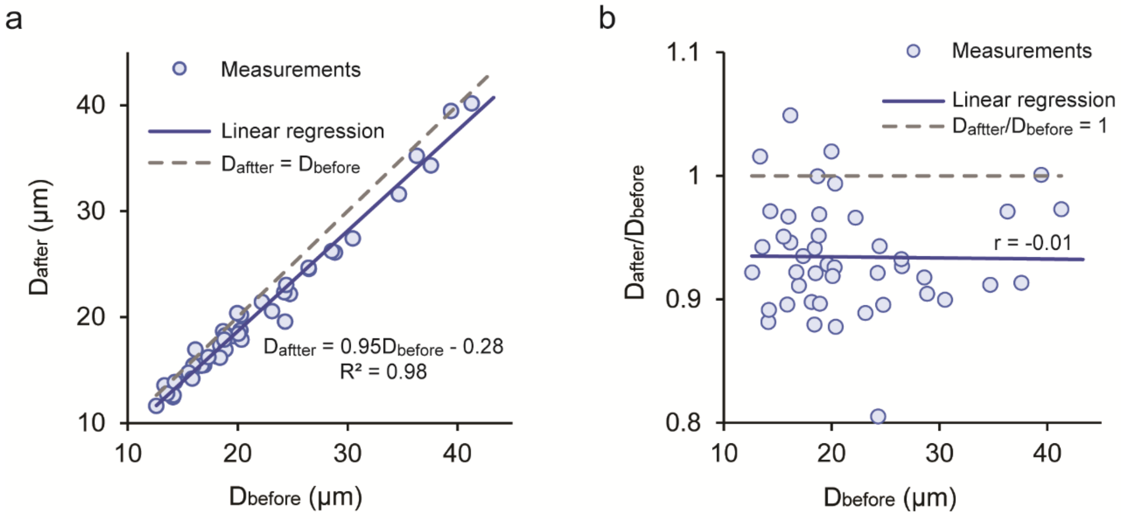

Supplementary Fig. 8 Radial constriction of lipid vesicles by FtsZ-ring. Scatter plots of the short axis of the deformed vesicle (D_{after}) (**a**) or the degree of deformation (D_{after}/D_{before}) (**b**) against diameter of vesicles before deformation (D_{before}). Plots (blue dots) were calculated among deformed vesicles (aspect ratio < 0.95) after 2-3 h co-expression of 5 essential genes at 37 °C. Graphs indicate the diameter of shorter axis of deformed vesicles (D_{after}) was smaller than original vesicle diameters (D_{before}), irrespective of vesicle size, showing that FtsZ-ring radially constricts lipid vesicles. n = 45. Source data are provided as a Source Data file.

Supplementary Table 1 List of primers.

Name	Sequence (5' to 3')
KN230	ATGGCACGCATTATTGTTGTTACTT
KN256	GGTATATCTCCTTCTTAAAGTTAAACA
932	AGACCACAACGGTTTCCCTCTAGAAATAATTTTGTTTAAC
PG43	TGAAACATGGCAAAGGTAGCGT
933	AAACCGTTGTGGTCTCCCTATAGTGAGTCGTATTAAT
PG44	GCTACCTTTGCCATGTTTCAGAAA
899	TTGCTCACCATGTGATGATGATGATGATGATGCCT
900	CTGTACAAGGGTGCAGCGGGTGAATTCA
227	ATGGTGAGCAAGGGCGAG
641	CTTGTACAGCTCGTCCATGC
885	TTCTTCGCCTTTGCTAACGTGATGATGATGATGATGATGCCTCAT
886	ATGGATGAACTGTATAAAGGTGCAGCGGGTGAATTCAGC
692	GTTAGCAAAGGCGAAGAAC
693	TTTATACAGTTCATCCATACCA
1065	GGTGCAGCGGGTGAATTC
979	GTGATGATGATGATGATGATGCCT
1063	CATCATCACATGGTTTCTAAAGGTGAGGCGGT
1064	CGCTGCACCTTTGTACAGTTCGTCCATACCGC
934	CTAGCATAACCCCTTGGG
510	CATGGTATATCTCCTTCTTAA
945	GATATACCATGATCAAGGCGACGGAC
946	GTTATGCTAGTTAAAACTCTTTTCGCAGCC
947	GAGATATACCATGATTAAAGCTACGGACAGAAAA
948	TTTTCTGTCCGTAGCTTTAATCATGGTATATCTC
949	GATTAAAGCTACTGATAGAAAACTGG
950	CCAGTTTTCTATCAGTAGCTTTAATC

Supplementary Table 1 List of primers (continued).

Name	Sequence (5' to 3')
951	GCTACTGATAGAAAATTAGTAGTAGGAC
952	GTCCTACTACTAATTTTCTATCAGTAGC
KN908	CATGGTATATCTCCTTCTTAAAGTTAAAC
KN170	ATGTTTGAACCAATGGAACTTAC
1051	TCCAACCGCTGTTTTACG
SC46	ATAAAGTTGCAGGACCACTTCTG
1050	CAGACGATTCAAATCGGTAGCG
SC47	CAGAAGTGGTCCTGCAACTTTAT
1052	GCGGTTGGAATGGTGAGCAAGGGCGAG
1053	AATCGTCTGCTTGTACAGCTCGTCCATGC
509	GCACTCGAGCACCACCAC
685	GGTATATCTCCTTCTTAAAGTTAAACAAAA
1021	TAAGAAGGAGATATACCATGAGCAAAGGAGAAGAACTTTTC
1022	TGGTGGTGCTCGAGTGCTCATTTGTAGAGCTCATCCATGCCAT
SC46	ATAAAGTTGCAGGACCACTTCTG
960	CCGCATCATTAGTTAATTCCATTGGTTC
SC47	CAGAAGTGGTCCTGCAACTTTAT
959	GAACCAATGGAATTAACTAATGATGCGG
1173	CGAGCTTAAAGACAGTAGCTTCA
1174	TGAAGCTACTGTCTTTAAGCTCG
T7P-F	CCCGCGAAATTAATACGACTCAC
T7P-R	CAAAAAACCCCTCAAGACCCGT

Supplementary Table 2 List of DNA templates for cell-free expression.

Linearized template	Original plasmid
sfGFP	pCoofy-sfGFP
ftsA	pET28a-FtsA
ftsAopt	pPT1-FtsAopt
minDEoperon	pPT1-MinD.MinE
mCherry-minC	pET28a-His-mCherry-MinC
ftsZopt-G55-Venus-Q56	pET11b-FtsZopt-G55-Venus-Q56
ftsZ-Venus	pET11b-FtsZ-Venus

Supplementary Table 3 List of DNA templates and concentrations for cell-free expression.

Condition	Templates and concentrations
Estimation of sfGFP expression level (Supplementary Fig. 4)	2 nM *sfGFP*
FtsA expression with FtsZ-Alexa488 (Fig. 3b, Supplementary Fig. 5b-e, and Supplementary Movie 3)	5 nM *ftsA*
Co-expression of FtsA, MinD, and MinE (Fig. 3c, d, Supplementary Fig. 5f, and Supplementary Movie 4)	2 nM *ftsAopt* and 2 nM *minDEoperon*
Co-expression of mCherry-MinC, MinD, and MinE (Supplementary Fig. 6b and Supplementary Movie 5)	2 nM *mCherry-MinC* and 1 nM *minDEoperon*
Estimation of wave emergence (Supplementary Fig. 6c)	0.5 nM *minDEoperon* with or without 1 nM *mCherry-MinC*
Co-expression of FtsA and FtsZ variants (Supplementary Fig. 6d, e, h, i, and Supplementary Movie 6)	1 nM *ftsAopt* and 3 nM *ftsZopt-G55-Venus-Q56* or *ftsZ-Venus*
Co-expression of FtsA, FtsZ-G55-Venus-Q56, mCherry-MinC, MinD, and MinE (Fig. 4b-f, Supplementary Fig. 7, 8, Supplementary Movie 7, 8, and 9)	1 nM *ftsAopt*, 3 nM *ftsZopt-G55-Venus-Q56*, 2 nM *mCherry-MinC*, and 1 nM *minDEoperon*

Supplementary Notes

Validation of MinCDE or FtsZ-mts reconstitution inside lipid vesicles under macromolecular crowding environments

In our experimental setup, we used fluorescently labeled MinC (mScarlet-I-MinC) to avoid any side effects in the Min wave dynamics as result of the fluorescent protein when it is fused to MinD or MinE. When the MinCDE system was encapsulated within lipid vesicles, they self-assembled into different patterns: Dynamic waves at the membrane (further categorized into previously described modes in spherical vesicles: Pole-to-pole oscillations, traveling waves (also referred as circling waves in several literatures[1-3], and pulsing) (Supplementary Fig. 2a), stationary localization at the membrane, and stationary localization in the lumen[3,4]. The emergence of such oscillatory patterns are strongly dependent on the MinDE ratios and concentration[4]. Therefore, spatiotemporal dynamics did not emerge when either MinD or MinE was in excess, resulting in stationary membrane localization at high MinD concentration or in the lumen at high MinE (Supplementary Fig. 2c and d). We further analyzed the relative frequency of different modes among conditions with predominant Min dynamics (>65%). Equimolar concentrations of MinDE at 3 µM favored the generation of traveling waves and pole-to-pole oscillations (Supplementary Fig. 2b). As pole-to-pole oscillations would be the desired mode for the spatiotemporal regulation of FtsZ at the membrane, we selected this MinDE concentration ratio as our standard condition for further assays.

Moreover, since previous studies stated that macromolecular crowding enhance the FtsZ polymerization[5-8] and sharpened the regulation of FtsZ by Min waves into steeper gradients[9-11], we figured that addition of certain macromolecular crowder agents in

solution might improve functional co-reconstitution of both systems. Thus, we encapsulated the Min system in presence of crowding by using Dextran 70 and Ficoll70 and observed that they were also functional. Without crowders, Min proteins had yielded the three dynamic modes; pole-to-pole oscillation, traveling waves, and pulsing, at roughly 15%: 35%: 18% ratios at our standard MinCDE conditions (equimolar concentration of MinDE at 3 μM and mScarlet-I-MinC at 0.5 μM). Intriguingly, after adding crowders, we observed a slight increase in the pole-to-pole oscillations, in spite of the unaltered spherical symmetry of the vesicle, which usually precludes mode selection[3]. Since stable pole-to-pole oscillations are required for correct placement of the FtsZ-ring *in vivo*[12,13], any improvement in the prevalence of this wave mode would be highly beneficial for the assembly and placement of a functional division ring. However, the increase in the frequencies was not highly significant at particular crowding conditions, reaching up to 29% of pole-to-pole oscillations at 100 g/L Ficoll70, for instance (Fig. 1e). Therefore, we concluded that macromolecular crowding did not significantly affect the frequency of Min patterns under our experimental conditions. However, we have not analyzed whether other features of Min wave dynamics were affected by crowding in solution such as interaction with the membrane, wavelength, velocity of the Min waves, or protein-protein interactions such as MinD-MinE complex to induce MinD's ATPase activity[9,14,15]. At the same time, we confirmed that a high percentage of the vesicles (70-80%) showed Min wave dynamics under all crowding conditions, thus supporting the co-reconstitution of Min waves together with FtsZ.

On the other hand, we have also validated the effects of macromolecular crowding environments on FtsZ-Venus-mts, an FtsZ mutant that contains the membrane

attachment sequence from MinD[16]. This mutant is able to bind the membrane independently of the interaction with the membrane anchor FtsA, which is challenging to reconstitute, due to their aggregative nature[17]. FtsZ-Venus-mts has greatly supported and facilitated the *in vitro* reconstitution and study of FtsZ on membranes without dramatically affecting its assembly dynamics[9,16,18-20]. Therefore, we have encapsulated FtsZ-Venus-mts in lipid vesicles under our experimental setup, and successfully observed the assembly of different structures upon GTP hydrolysis and addition of macromolecular crowders (Fig. 1b and Supplementary Fig. 1a). The higher amount of FtsZ bundles at higher crowding conditions determined that macromolecular crowding is required to functionally connect the FtsZ and MinCDE system.

Effects of macromolecular crowding on PURE Cell-free expression

Before we started to reconstitute the Min or FtsZ-FtsA system with the cell-free expression system, we first tested the effects of crowding on our experimental setup by expression of sfGFP. Compared to the expression without crowders, we confirmed a relatively lower expression yield of sfGFP under crowding environments, as previously reported[22-25] (Supplementary Fig. 4). However, the sfGFP yield reached about 10 µM with Ficoll70 after 200 min of cell-free expression, whereas Dextran70 radically impaired the sfGFP expression level (Supplementary Fig. 4g). Because MinCDE, FtsZ and FtsA proteins would need up to 5 µM of the expression yield, we decided to employ the Ficoll70 as a crowding agent in cell-free expression experiments.

Analysis of reconstituted MinCDE waves via cell-free expression

After we reconstituted MinCDE waves via co-expression of mCherry-MinC, MinD, and

MinE, we further characterized the Min wave dynamics emerged by cell-free expressed proteins. The kymographs revealed that Min wave patterns gradually appeared along with the synthesis of mCherry-MinC (Supplementary Fig. 6b). As for MinDE co-expression (Fig. 4c and Supplementary Fig. 5f), wave patterns dynamically transited from pole-to-pole oscillation to traveling waves. Moreover, extended time-lapse observation up to 2.5 h captured that Min waves eventually showed static patterns, which agreed with the emergence of static patterns after 2-4 h of MinDE expression[26]. Then, we analyzed the time-dependent occurrence of Min wave patterns inside lipid vesicles via cell-free expression system. The efficiency of Min patterns (dynamic waves and static patterns) among vesicles were lower and also slower in full MinCDE co-expression system compared to MinDE expression, (Supplementary Fig. 6c). This impaired efficiency of wave emergence might be due to the conflict of the protein synthesis resources between mCherry-MinC and MinDE templates. However, we confirmed that at least 60% of the vesicles contained Min patterns after 1 h co-expression of MinCDE proteins.

Functional FtsZ-FP for FtsZ-FtsA reconstitution via cell-free expression

In this study, we employed FtsZ-G55-Venus-Q56 to visualize a functional FtsZ using fluorescence microscopy. It is previously known that the C-terminal conjugation with FPs impairs the functionality of FtsZ *in vivo*[27,28], because interactions with division proteins occur mainly through the C-terminal domain of FtsZ[29,30]. At the same time, the considerable gain in molecular weight from the fusion of FPs with wild-type FtsZ (FtsZ-FPs, ~67 kDa) renders it difficult to implement a close-to-physiological concentration of FtsZ (1-6 μM)[31-33] using cell-free expression with respect to FtsA (45 kDa, 0.07-0.3

μM)[32,33].

To overcome these difficulties, we tested a FtsZ-G55-FP-Q56 mutant, in which a fluorescent protein was inserted in between 55th Gly and 56th Gln FtsZ residues according to a previous report[34]. As one of those chimeras (FtsZ-G55-mVenus-Q56) was reported as fully functional *in vivo*, it was naturally expected to have similar functionalities as wild-type FtsZ *in vitro*. Together with the validation of template concentration ratio (3 nM for FtsZ-G55-Venus-Q56 and 1 nM for FtsA), we observed the FtsZ mesh structure on the membrane (Supplementary Fig. 6d and e). On the other hand, C-terminally fused FtsZ-Venus did not show preferential membrane localization, verifying that this chimeric FtsZ protein lacks this functionality (Supplementary Fig. 6d and e). Moreover, we checked the activity of purified FtsZ-G55-Venus-Q56 on SLBs and by GTPase assay, confirming that FtsZ-G55-Venus-Q56 is active under our experimental conditions and it is able to form dynamic structures on SLBs (Supplementary Fig. 6f and g).

Intriguingly, we observed a size dependency of FtsZ intensity on the membrane among both FtsA-only and FtsZ-FtsA expression system. In case of FtsA expression with purified FtsZ-Alexa488, the localization of FtsZ molecules in the lumen of the vesicle was gradually decreased by the expression of FtsA (Supplementary Fig. 5b), while an increase of FtsZ intensity was observed on the membrane (Supplementary Fig. 5d). Furthermore, higher FtsZ concentrations on the membrane were observed in larger vesicles, suggesting a correlation between size and membrane coverage of FtsZ (Supplementary Fig. 5e). Since surface-to-volume ratio decreases in larger vesicles, this result suggests that the majority of FtsZ molecules sufficiently bound to the membrane through FtsA expression regardless the vesicle sizes. In parallel, FtsA-FtsZ co-

expression system also indicated gradual increase of the FtsZ intensity on membrane along with cell-free expression (Supplementary Fig. 6h) and relatively higher FtsZ level within larger vesicles (Supplementary Fig. 6h and i). This size dependency also supports the increase of the concentration of FtsZ on the membrane among relatively larger vesicles.

Min wave organized FtsZ structures in co-expression system

After we reconstituted co-expression system of MinCDE and FtsZ-FtsA proteins (Fig. 4), we characterized the time-dependent FtsZ dynamics regulated by Min waves inside lipid vesicles. In some cases, time-lapse images suggested that Min waves first appeared as pole-to-pole oscillations to form the FtsZ-ring, and later transformed into a static bipolar localization depending on MinDE concentrations (Fig. 4c and Supplementary Fig. 7a). Surprisingly, although we suspect that some sort of energy-driven dynamic mechanism is at place in these vesicles, the spatial regulation of the FtsZ-ring like structure to the middle of the vesicles seems to be preserved without pole-to-pole oscillation of Min waves (Fig. 4c and Supplementary Fig. 7a). The circumferential (360°) surface plot of FtsZ intensities revealed that FtsZ was first randomly distributed on the membrane but then formed and reinforced two bands over time, corresponding to a ring-like structure and implying that the Min waves successfully reshaped FtsZ structures and stabilized them (Supplementary Fig. 7c). In addition, also non-deformed vesicles showed FtsZ mesh structures antagonistically localized against Min traveling waves (Supplementary Fig. 7b). The fact that all three wave modes (pole-to-pole oscillation, bipolar static localization, and traveling waves) could be observed after MinCDE expression might reflect differences of MinDE concentrations among vesicles (Supplementary Fig. 6b).

Supplementary Discussion

In this work we have achieved the *in vitro* reconstitution of a minimal division machinery composed by FtsZ-ring located at the equatorial plane of a lipid cell-like container by the dynamic MinCDE system, reassembling their behavior inside the cell. It might represent the first step towards the reconstitution of a functional division machinery able to fulfill division of cell-like systems in vitro, one of the major goals of the bottom-up Synthetic biology. Moreover, as mentioned in the main text, the use of two different approaches in this work has allowed us not only the characterization of our experimental setup at higher degree, but also revealing some of the key factors involved in the process (such as protein concentration, protein ratios, crowding conditions and buffer composition). Thus, by using these two approaches, we provided higher robustness to our results and helpful improvements for the *in vitro* reconstitution of protein systems in cell-like platforms.

Although co-reconstitution of the MinCDE system and FtsZ-Venus-mts has been performed previously[1,10,11], experimental setups used in those studies lack some critical features as a cell model, namely, fully confined spaces and deformable membrane surface, where both features have significant impacts over the FtsZ and MinCDE protein dynamics. For instance, it is known that cell-sized confinement significantly alters the requirements to induce Min waves[14], and also deformable membrane systems play an essential role to study the FtsZ dynamics[20,21,35,36]. For instance, Zieske and Schwille[10] reconstituted the Min pole-to-pole oscillation together with FtsZ-mts condensation into the middle of PDMS fabricated chamber in particular, however, this demonstration was far away from the reconstitution of those protein systems inside cell models. Taken together, our observation presented in this study has firstly reported the formation and

assembly of a FtsZ-ring located at the middle of the lipid vesicles regulated by the MinCDE waves.

One of the key features of our work is optimization of macromolecular crowding environments to achieve the assembly of the FtsZ-ring. Macromolecular crowding in solution non-specifically enhances molecular interactions by a volume exclusion effect that have a huge potential to modulate the kinetics and equilibria of a large number of molecular reactions taking place inside the cell[37,38]. The effect of macromolecular crowding in solution also influence the protein-membrane interactions since they are thermodynamically and kinetically linked. Eventually, any volume exclusion effect over the molecules in solution will affect their equilibria and interaction with the membrane as well. To study these molecular dynamics, synthetic macromolecules like Dextran, Ficoll, or PEG as well as proteins like BSA have been extensively used to mimic the crowded cellular environment *in vitro*[39-41]. However, a repulsive interaction of PEG with other molecules cannot be fully described quantitatively by an effect of excluded volume alone, involving certain attractive interaction between PEG and the hydrophobic side chains on the protein surface[37,40,41]. For this reason, we have decided to exclude PEG from our studies.

Previous studies have demonstrated the significant impacts of macromolecular crowding over FtsZ and MinCDE protein functionalities. Briefly, crowding conditions promote FtsZ bundling in solution and inside vesicles, increase oligomerization and promote the assembly of liquid-liquid phase-separated droplets[5,7,21,22,35,42,43]. On the other hand, macromolecular crowding decreases the wavelength and velocity of MinDE waves[9,15],

while enhancing the emergence of waves inside lipid microdroplets[14]. Thus, we found that macromolecular crowding becomes essential to reconstitute the minimal division system. Even though the emergence of Min pole-to-pole oscillations was only slightly increased under crowding conditions (Fig. 1e), the presence of FtsZ effectively increased the pole-to-pole oscillations for the case of the MinC (from 20% to 40% at 100 g/L Dextran70) in comparison with MinC^{G10D} mutant (From 25% to 31% at 100 g/L Dextran 70) (Fig. 1e and Fig. 2g). In addition, we have also observed a higher increment of FtsZ-ring formation with MinC under 100 g/L Dextran70 crowding condition (~42%), while absence of MinC or MinC^{G10D} mutant could only promote significantly less FtsZ-rings (~10-12%) (Fig. 2f). Since frequency of pole-to-pole oscillations was slightly increased even with MinC^{G10D} mutant, it is plausible to suggest that the FtsZ bundles on the membrane at high crowding conditions is one of the key factors to enhance pole-to-pole oscillation in cellular compartments. However, considering increment of the frequency of both pole-to-pole oscillation and FtsZ-ring formation with wild-type MinC, the formation of FtsZ-ring might have higher impact on enhancement of pole-to-pole oscillations following a mutual positive feedback with the Min waves.

Related to this, the mutual positive feedback between FtsZ-ring formation and pole-to-pole oscillation of Min waves suggested by our data is a new insight that was not expected by the current understanding of the molecular dynamics of those protein systems. However, it is also plausible to assume that this phenomenon is closely related to the geometrical effects on Min waves described previously. Essentially, Min waves can sense geometry and reorganize the wave patterns according to the shape of membrane matrix or physical barriers[10,15,44]. In this regard, considering the presence of

thick FtsZ bundles on the membrane promoted by crowding, they might behave as a physical barrier, limiting Min proteins to access to the successive membrane area required to emerge traveling waves. Indeed, the presence of FtsZ bundles slightly increased the pole-to-pole oscillations (From 25% to 31%) without effective regulation of Min waves in case of $MinC^{G10D}$ mutant (Fig. 1e and Fig. 2g). Taken together, our observations point to a critical biological relevance of macromolecular crowding in the synergetic dynamics among Min waves, FtsZ bundles, FtsZ-ring structures, and environmental factors such as membrane interaction and confinement. We are only at the beginning of appreciating the mutual relationships of these factors to decipher their roles inside cells.

In order to further improve our experimental setup and eventually achieve the full constriction of the lipid vesicles by the FtsZ-ring together with other division-related proteins, several approaches would be considered in future studies. One of the most intriguing suggestion from our study is the higher deformability observed in bigger vesicles in case of the FtsZ-FtsA system, finding a size dependence of vesicle deformation by FtsZ-rings (Fig. 4f and Supplementary Fig. 7). Since the surface-to-volume ratio becomes smaller in larger spheres, FtsZ-FtsA density on the membrane might be increased for larger vesicles at the same protein expression level, as shown in our analysis (Supplementary Fig. 4e, 5h, i). Higher density of FtsZ-FtsA on the membrane would explain why significant deformations can be only observed in larger vesicles, suggesting that higher concentration of FtsZ and FtsA proteins on the membrane might be able to exert greater force, highlighting the importance of protein density on the membrane. Besides, size-dependent mechanical properties of vesicle

membrane such as surface tension and stiffness would favor higher deformation in larger vesicles[45-47]. Quantitative biophysical measurements, e.g., by micropipette aspiration and/or optical tweezers should be carried out in future studies to reveal the induced forces and clarify the key features driving vesicle deformation among a broad range of vesicle sizes. In addition, the lack of major deformations using the FtsZ-Venus-mts (Fig. 2b-e and Supplementary Fig. 3) remarked the influence of different membrane attachment, which could be a critical factor for the generation of greater forces on the membrane, as it can modulate the treadmilling behavior of FtsZ[18-20]. Thus, the control of the protein density on the membrane together with different FtsZ attachment protein such as ZipA might be an interesting approach to improve the force generation by a FtsZ-ring and therefore deformation of vesicles.

Next, our experimental setup using minimal division proteins can serve as a platform to increase the complexity of the system adding more components such as the Zap proteins, which can enhance the bundling of FtsZ[48], the nucleoid occlusion system to reinforce the displacement of FtsZ from the lumen[49], or ZipA as a linker of the FtsZ protein to the membrane. The addition of complementary division components is an attractive approach to improve the system and achieve the reconstitution of a functional divisome *in vitro*. In this regard, ZipA can be a good candidate to improve the deformations by a FtsZ-ring as we discussed in the main text. Since ZipA contains a transmembrane region instead of a membrane binding domain as FtsA or MinD[50] and its insertion into membrane bilayer might be able to enhance the membrane bending as hypothesized previously[51], the combination of FtsZ and ZipA might induce further deformations of membrane by FtsZ torsion and treadmilling. Hence, co-reconstitution of FtsZ, FtsA, and ZipA as a proto-ring together with Min proteins can potentially improve the current system

leading to a higher degree of membrane deformation.

Lastly, the study of different membrane properties and their impact in the formation of the FtsZ-ring formation and deformation of vesicles is an interesting approach to improve the system. For instance, crowding at the membrane is an interesting approach to enhance the polymerization dynamics of FtsZ, as it was previously shown by using bacterial cytoskeleton protein MreB[52]. Different PEG derivatives linked to the membrane[52] can be a good candidate together with different lipid compositions to obtain further optimized membrane properties such as charge, rigidity, surface tension, diffusion, and phase-separation. Some of these properties have been already stated that affect the behavior of MinCDE and FtsZ[31] and therefore, can potentially improve and modulate the membrane properties in order to achieve higher frequencies of ring formation, membrane deformability or even full scission of the membrane.

Supplementary Methods

Plasmid construction

The basic information for plasmid construction is given in methods in the main manuscript. However, again, plasmids were typically constructed using seamless cloning. DNA fragments were amplified with overlaps regions between adjacent fragments by PCR, and then PCR products were treated with DpnI and combined using GeneArt Seamless Cloning and Assembly Enzyme Mix. For deletion of sequences from plasmids, plasmids were constructed using blunt end cloning. A DNA fragment was amplified from the original plasmid, treated with DpnI, phosphorylated using T4 Phosphokinase, and then ligated with T4 DNA Ligase. All enzymes for cloning were purchased from Thermo Fisher Scientific (Waltham, MA, USA). Detailed methods of each plasmid are described below, and primers are listed in Supplementary Table 1.

pIVEX2.3d-sfGFP-His for purification of sfGFP was a kind gift from Dr. Lei Kai from the School of Life Sciences, Jiangsu Normal University, China. A DNA fragment for pCoofy backbone was amplified from pCoofy1 (Addgene plasmid # 43974) with a 509/685 primer set, then, *sfGFP* gene was amplified from pIVEX2.3d-sfGFP-His with a 1021/1022 primer set. These PCR products were assembled using seamless cloning.

pET28a-His-msfGFP-MinC, pET28a-His-mCherry-MinC, and pET28a-His-mScarlet-I-MinC were constructed by substituting the *eGFP* gene from pET28a-His-eGFP-MinC[53] by genes encoding respective fluorescent proteins. First, pET28a backbone was amplified from pET28a-His-eGFP-MinC with an 885/886 primer set. Then, *msfGFP* gene was amplified from pET28a-His-MinE (2-31)-msfGFP[54] with a 692/693 primer set. These PCR products were assembled using seamless cloning to construct pET28a-His-msfGFP-MinC. For pET28a-His-mCherry-MinC, pET28a backbone was amplified in the

same manner with an 899/900 primer set. Then, *mCherry* gene was amplified from pLVX_mCherry-C1 (Clontech, Mountain View, CA, USA) with a 641/227 primer set. These PCR products were assembled using seamless cloning. For pET28a-His-mScarlet-I-MinC, pET28a backbone was similarly amplified from pET28a-His-eGFP-MinC with a 1065/979 primer set. *mScarlet-I* gene sequence was optimized for the *E. coli* expression system and synthesized as a DNA cassette (Thermo Fisher Scientific). *mScarlet-I* DNA cassette was then amplified with a 1063/1064 primer set and assembled with a pET28a backbone using seamless cloning.

To construct pPT1-MinD.MinE plasmid, we first deleted the His-tag from pET28a-His-MinD.MinE[55] to obtain *minD-minE* operon sequence without any tag sequences. A part of pET28a-His-MinD.MinE was amplified by PCR with a KN230/KN256 primer set, and subsequently, the PCR product was conjugated using blunt end cloning. The resultant plasmid (pET28a-MinD.MinE) was further optimized for the cell-free expression system by substituting the lac operator region with a stem-loop sequence. Two DNA fragments were amplified from pET28a-MinD.MinE using 932/PG43 and 933/PG44 sets, respectively. Then, PCR products were assembled using seamless cloning. The resultant plasmid was named pPT1-MinD.MinE since the plasmid is not suitable for protein expression like a typical pET system because of the lack of the lac operator region.

pPT1-FtsAopt was constructed by cloning the *ftsA* gene into pPT1-MinD.MinE and following optimization of *ftsA* gene for cell-free expression system. pPT1 plasmid backbone was amplified from pPT1-MinD.MinE with a 934/510 primer set. Then, *ftsA* gene was amplified from pET28a-FtsA[56] with a 945/946 primer set, and those PCR products were assembled using seamless cloning. The resultant plasmid (pPT1-FtsA)

was further optimized to increase the AT-content in the first 30 DNA sequences (10 Amino acids) by introducing silent point mutations[2,22,35]. Then, the point mutations (substitution of 7 nucleotides) were introduced in three repeats of mutagenesis. First, two fragments were amplified from pPT1-FtsA using 947/PG43 and 948/PG44 primer sets, respectively. These fragments were assembled using seamless cloning to construct the intermediate plasmid (pPT1-FtsA_3xMut). Then, the same procedure was repeated to introduce further mutations with 949/PG43 and 950/PG44 primer sets (obtained the intermediate plasmid, pPT1-FtsA_5xMut). Finally, the rest of the mutations were introduced using 951/PG43 and 952/PG44 primer sets to obtain pPT1-FtsAopt.

pET11b-FtsZ-Venus (conjugation of Venus in the C-terminus of full-length FtsZ) was obtained from pET11b-mts-FtsZ-Venus (Venus was referred as YFP in the previous manuscripts)[18,57] by removing the mts region. A DNA fragment was amplified from pET11b-mts-FtsZ-Venus using a KN908/KN170 primer set and conjugated using blunt end cloning.

pET11b-FtsZ[58] was first optimized for cell-free expression similar to FtsA optimization, and then *Venus* gene was inserted between G55 and Q56 amino acid residue of FtsZ to obtain pET11b-FtsZopt-G55-Venus-Q56. Two DNA fragments were amplified from pET11b-FtsZ using SC46/960 and SC47/959 primer sets. These fragments were assembled using seamless cloning. The resultant plasmid (pET11b-FtsZopt) was linearized into two parts using 1051/SC46 and 1050/SC47 primer sets, respectively. Then, *Venus* gene fragment was obtained from pET11b-FtsZ-YFP-mts[16] using a 1052/1053 primer set. These three DNA fragments were then assembled using seamless cloning.

pET28a-His-mScarlet-I-MinC^{G10D} mutant[10] was constructed by substituting the Gly10

residue of *minC* gene by Asp from pET28a-His-mScarlet-I-MinC. Two DNA fragments with G10D mutation were amplified from pET28a-His-mScarlet-I-MinC with PG43/1173 and PG44/1174 primer sets and then these PCR products were assembled using seamless cloning to obtain pET28a-His-mScarlet-I-MinC^{G10D}.

Supplementary References

1. Zieske, K., Chwastek, G. & Schwille, P. Protein patterns and oscillations on lipid monolayers and in microdroplets. *Angew. Chemie Int. Ed.* **55**, 13455–13459 (2016).
2. Godino, E. et al. De novo synthesized Min proteins drive oscillatory liposome deformation and regulate FtsA-FtsZ cytoskeletal patterns. *Nat. Commun.* **10**, 4969 (2019).
3. Litschel, T., Ramm, B., Maas, R., Heymann, M. & Schwille, P. Beating vesicles: Encapsulated protein oscillations cause dynamic membrane deformations. *Angew. Chemie Int. Ed.* **57**, 16286–16290 (2018).
4. Kohyama, S., Fujiwara, K., Yoshinaga, N. & Doi, N. Conformational equilibrium of MinE regulates the allowable concentration ranges of a protein wave for cell division. *Nanoscale* **12**, 11960–11970 (2020).
5. Robles-Ramos, M. Á. et al. Assembly of bacterial cell division protein FtsZ into dynamic biomolecular condensates. *Biochim. Biophys. Acta - Mol. Cell Res.* **1868**, 118986 (2021).
6. Rivas, G., Fernández, J. A. & Minton, A. P. Direct observation of the enhancement of noncooperative protein self-assembly by macromolecular crowding: indefinite linear self-association of bacterial cell division protein FtsZ. *Proc. Natl. Acad. Sci. USA* **98**, 3150–3155 (2001).
7. González, J. M. et al. Essential cell division protein FtsZ assembles into one monomer-thick ribbons under conditions resembling the crowded intracellular environment. *J. Biol. Chem.* **278**, 37664–37671 (2003).
8. Groen, J. et al. Associative interactions in crowded solutions of biopolymers

counteract depletion effects. *J. Am. Chem. Soc.* **137**, 13041–13048 (2015).

9. Martos, A. et al. FtsZ polymers tethered to the membrane by ZipA are susceptible to spatial regulation by Min waves. *Biophys. J.* **108**, 2371–2383 (2015).
10. Zieske, K. & Schwille, P. Reconstitution of self-organizing protein gradients as spatial cues in cell-free systems. *eLife* **3**, e03949 (2014).
11. Ramm, B. et al. The MinDE system is a generic spatial cue for membrane protein distribution in vitro. *Nat. Comm.* **9**, 3942 (2018).
12. Raskin, D. M. & de Boer, P. A. J. Rapid pole-to-pole oscillation of a protein required for directing division to the middle of Escherichia coli. *Proc. Natl. Acad. Sci. USA* **96**, 4971–4976 (1999).
13. Hu, Z. & Lutkenhaus, J. Topological regulation of cell division in Escherichia coli involves rapid pole to pole oscillation of the division inhibitor MinC under the control of MinD and MinE. *Mol. Microbiol.* **34**, 82–90 (1999).
14. Kohyama, S., Yoshinaga, N., Yanagisawa, M., Fujiwara, K. & Doi, N. Cell-sized confinement controls generation and stability of a protein wave for spatiotemporal regulation in cells. *eLife* **8**, e44591 (2019).
15. Schweizer, J. et al. Geometry sensing by self-organized protein patterns. *Proc. Natl. Acad. Sci. USA* **109**, 15283–15288 (2012).
16. Osawa, M., Anderson, D. E. & Erickson, H. P. Reconstitution of contractile ftsZ rings in liposomes. *Science* **320**, 792–794 (2008).
17. Martos, A. et al. Isolation, Characterization and Lipid-Binding Properties of the Recalcitrant FtsA Division Protein from Escherichia coli. *PLoS ONE* **7**, e39829 (2012).

18. Ramirez-Diaz, D. A. *et al.* Treadmilling analysis reveals new insights into dynamic FtsZ ring architecture. *PLOS Biol.* **16**, e2004845 (2018).

19. García-Soriano, D. A., Heermann, T., Raso, A., Rivas, G. & Schwille, P. The speed of FtsZ treadmilling is tightly regulated by membrane binding. *Sci. Rep.* **10**, 10447 (2020).

20. Ramirez-Diaz, D. A. et al. FtsZ induces membrane deformations via torsional stress upon GTP hydrolysis. *Nat. Comm.* **12**, 3310 (2021).

21. Osawa, M. & Erickson, H. P. Liposome division by a simple bacterial division machinery. *Proc. Natl. Acad. Sci. USA* **110**, 11000–11004 (2013).

22. Furusato, T. et al. De novo synthesis of basal bacterial cell division proteins FtsZ, FtsA, and ZipA inside giant vesicles. *ACS Synth. Biol.* **7**, 953–961 (2018).

23. Norred, S. E. et al. Macromolecular crowding induces spatial correlations that control gene expression bursting patterns. *ACS Synth. Biol.* **7**, 1251–1258 (2018).

24. Ge, X., Luo, D. & Xu, J. Cell-free protein expression under macromolecular crowding conditions. *PLoS One* **6**, e28707 (2011).

25. Takahashi, K., Sato, G., Doi, N. & Fujiwara, K. A relationship between ntp and cell extract concentration for cell-free protein expression. *Life* **11**, 237 (2021).

26. Yoshida, A., Kohyama, S., Fujiwara, K., Nishikawa, S. & Doi, N. Regulation of spatiotemporal patterning in artificial cells by a defined protein expression system. *Chem. Sci.* **10**, 11064–11072 (2019).

27. Ma, X., Ehrhardt, D. W. & Margolin, W. Colocalization of cell division proteins FtsZ and FtsA to cytoskeletal structures in living Escherichia coli cells by using green fluorescent protein. *Proc. Nat. Acad. Sci. USA* **93**, 12998–13003 (1996).

28. Osawa, M. & Erickson, H. P. Probing the domain structure of FtsZ by random truncation and insertion of GFP. *Microbiology* **151**, 4033–4043 (2005).

29. Buske, P. J. & Levin, P. A. A flexible C-terminal linker is required for proper FtsZ assembly in vitro and cytokinetic ring formation in vivo. *Mol. Microbiol.* **89**, 249–263 (2013).

30. Ortiz, C., Natale, P., Cueto, L. & Vicente, M. The keepers of the ring: Regulators of FtsZ assembly. *FEMS Microbiol. Rev.* **40**, 57–67 (2015).

31. Ramm, B., Heermann, T. & Schwille, P. The E. coli MinCDE system in the regulation of protein patterns and gradients. *Cell. Mol. Life Sci.* **76**, 4245–4273 (2019).

32. Rueda, S., Vicente, M. & Mingorance, J. Concentration and assembly of the division ring proteins FtsZ, FtsA, and ZipA during the Escherichia coli cell cycle. *J. Bacteriol.* **185**, 3344–3351 (2003).

33. Schmidt, A. et al. The quantitative and condition-dependent Escherichia coli proteome. *Nat. Biotechnol.* **34**, 104–110 (2016).

34. Moore, D. A., Whatley, Z. N., Joshi, C. P., Osawa, M. & Erickson, H. P. Probing for binding regions of the FtsZ protein surface through site-directed insertions: Discovery of fully functional FtsZ-fluorescent proteins. *J. Bacteriol.* **199**, e00553-16 (2017).

35. Godino, E. et al. Cell-free biogenesis of bacterial division proto-rings that can constrict liposomes. *Commun. Biol.* **3**, 539 (2020).

36. Ganzinger, K. A. et al. FtsZ reorganization facilitates deformation of giant vesicles in microfluidic traps. *Angew. Chemie Int. Ed.* **132**, 21556–21560 (2020).

37. Zhou, H.-X., Rivas, G. & Minton, A. P. Macromolecular crowding and confinement: biochemical, biophysical, and potential physiological consequences. *Annu. Rev. Biophys.* **37**, 375–397 (2008).

38. Rivas, G. & Minton, A., Influence of Nonspecific Interactions on Protein Associations: Implications for Biochemistry In Vivo. *Annu. Rev. Biochem.* **91**, 5.1–5.31 (2022).

39. Rivas, G., Fernández, J. A. & Minton, A. P. Direct observation of the enhancement of noncooperative protein self-assembly by macromolecular crowding: indefinite linear self-association of bacterial cell division protein FtsZ. *Proc. Natl. Acad. Sci. USA* **98**, 3150–3155 (2001).

40. Minton, A. P. The effect of volume occupancy upon the thermodynamic activity of proteins: some biochemical consequences. *Mol. Cell Biochem.* **55**, 119–140 (1983).

41. Winzor, D. J. & Wills, P. R. Molecular crowding effects of linear polymers in protein solutions. *Biophys. Chem.* **119**, 186–195 (2006).

42. Cabré, E. J. et al. Bacterial division proteins FtsZ and ZipA induce vesicle shrinkage and cell membrane invagination. *J. Biol. Chem.* **288**, 26625–26634 (2013).

43. Monterroso, B., Reija, B., Jiménez, M., Zorrilla, S. & Rivas, G. Charged Molecules Modulate the Volume Exclusion Effects Exerted by Crowders on FtsZ Polymerization. *PLoS ONE* **11**, e0149060 (2016).

44. Wu, F., Van Schie, B. G. C., Keymer, J. E. & Dekker, C. Symmetry and scale orient Min protein patterns in shaped bacterial sculptures. *Nat. Nanotechnol.* **10**, 719–726 (2015).

45. Lin, C. M., Li, C. S., Sheng, Y. J., Wu, D. T. & Tsao, H. K. Size-dependent properties of small unilamellar vesicles formed by model lipids. *Langmuir* **28**, 689–700 (2012).

46. Lipowsky, R. Remodeling of membrane shape and topology by curvature elasticity and membrane tension. *Adv. Biol.* **6**, 2101020 (2021).

47. Dimova, R. & Marques, C. *The Giant Vesicle Book*. (CRC Press, 2019).

48. Durand-Heredia, J., Rivkin, E., Fan, G., Morales, J. & Janakiraman, A. Identification of ZapD as a cell division factor that promotes the assembly of FtsZ in Escherichia coli. *J. Bacteriol.* **194**, 3189–3198 (2012).

49. Tonthat, N.K., Milam, S.L., Chinnam, N., Whitfill, T., Margolin, W. & Schumacher, M. A. SlmA forms a higher-order structure on DNA that inhibits cytokinetic Z-ring formation over the nucleoid. *Proc. Natl. Acad. Sci. USA* **110**, 10586–10591 (2013).

50. Moy, F. J., Glasfeld, E., Mosyak, L. & Powers, R. Solution Structure of ZipA, a Crucial Component of Escherichia coli Cell Division. *Biochemistry* **39**, 9146–9156 (2000).

51. Derganc, J. & Čopič, A. Membrane bending by protein crowding is affected by protein lateral confinement. *Biochim. Biophys. Acta - Biomembr.* **1858**, 1152–1159 (2016).

52. Garenne, D., Libchaber, A. & Noireaux, V. Membrane molecular crowding enhances MreB polymerization to shape synthetic cells from spheres to rods. *Proc. Natl. Acad. Sci. USA* **117**, 1902-1909 (2020).

53. Loose, M., Fischer-Friedrich, E., Herold, C., Kruse, K. & Schwille, P. Min protein patterns emerge from rapid rebinding and membrane interaction of MinE. *Nat.*

Struct. Mol. Biol. **18**, 577–583 (2011).

54. Glock, P., Brauns, F., Halatek, J., Frey, E. & Schwille, P. Design of biochemical pattern forming systems from minimal motifs. *eLife* **8**, e48646 (2019).

55. Loose, M., Fischer-Friedrich, E., Ries, J., Kruse, K. & Schwille, P. Spatial regulators for bacterial cell division self-organize into surface waves in vitro. *Science* **320**, 789–792 (2008).

56. Geissler, B., Elraheb, D. & Margolin, W. A gain-of-function mutation in ftsA bypasses the requirement for the essential cell division gene zipA in Escherichia coli. *Proc. Natl. Acad. Sci. USA* **100**, 4197–4202 (2003).

57. Osawa, M. & Erickson, H. P. Inside-out Z rings - constriction with and without GTP hydrolysis. *Mol. Microbiol.* **81**, 571–579 (2011).

58. Bramhill, D. & Thompson, C. M. GTP-dependent polymerization of Escherichia coli FtsZ protein to form tubules. *Proc. Nati. Acad. Sci. USA* **91**, 5812–5817 (1994).
