# Supplementary material for: In vitro assembly, positioning and contraction of a division ring in minimal cells
Source: Nat Commun. 2022 Oct 15;13:6098. doi: 10.1038/s41467-022-33679-x (PMC9569390; doi:10.1038/s41467-022-33679-x)
Supplement: Supplementary file 3 — Description of Additional Supplementary Files [file 41467_2022_33679_MOESM3_ESM.pdf]

Description of Additional Supplementary Files

Note: in all movies, Z-stack of fluorescence images were reconstituted in 3D max projection, and MinC variants and FtsZ variants are indicated in magenta (MinC) or green (FtsZ), respectively.

File name: Supplementary Movie 1

Description: MinCDE waves inside lipid vesicles in the presence of 100 g/L of Ficoll70. 3 µM His-MinD, 3 µM MinE-His, and 0.5 µM mScarlet-I-MinC were encapsulated inside lipid vesicles together with 2.5 mM ATP and crowding agent. Min proteins self-assemble following different patterns on the membrane. Time indicates mm:ss. Scale bar: 5 µm.

File name: Supplementary Movie 2

Description: Formation of an FtsZ-ring structure driven by the MinCDE wave in the presence of macromolecular crowding using 50 g/L Dextran70. A single bundle formed by FtsZ filaments was formed by the emergence of the Min wave pole-to-pole oscillation. Thus, this FtsZ-ring structure is continuously repositioned around the equatorial plane of the vesicle by its antagonistic localization with the MinCDE protein wave. Both systems demonstrated a synergic effect as positive feedback to each other, being stable for long times. Time indicates mm:ss. Scale bar: 15 µm.

File name: Supplementary Movie 3

Description: FtsZ mesh formation inside lipid vesicles via cell-free expression of FtsA with FtsZ-Alexa488. FtsZ indicated dynamic assembly into mesh structures on the membrane via FtsA expression under the Ficoll70 crowding environment. Recording was started after 20 min incubation at 37 °C. Time indicates hh:mm:ss. Scale bar: 10 µm.

File name: Supplementary Movie 4

Description: Formation of FtsZ-ring structure driven by Min waves within lipid vesicles via co-

expression of FtsA, MinD, and MinE with purified FtsZ-Alexa488 and mCherry-MinC. Transition of FtsZ structures was strongly regulated by Min wave patterns. Initially, FtsZ and Min proteins indicated no patterns, and then FtsZ formed mesh structure on the membrane via expression of FtsA similar to the Supplementary Movie3. Lately, an FtsZ-ring structure emerged together with pole-to-pole oscillations of Min waves and then settled to antagonistic localization of Min traveling waves and FtsZ mesh. Recording was started after 10 min incubation at 37 °C. Time indicates hh:mm:ss. Scale bar: 10 μm.

File name: Supplementary Movie 5

Description: Min wave emergence inside lipid vesicles through co-expression of mCherry-MinC, MinD, and MinE. mCherry signal is faint at the beginning of the recording because of low expression yield of mCherry-MinC, however, Cell-free expressed Min proteins successfully emerged dynamic Min waves and static patterns after 30 min of recording. Recording was started after 30 min incubation at 37 °C. Time indicates hh:mm:ss. Scale bar: 20 μm.

File name: Supplementary Movie 6

Description: FtsZ mesh development by cell-free expressed FtsA and FtsZ-G55-Venus-Q56 within lipid vesicles. Like Supplementary Movie3, FtsZ-G55-Venus-Q56 formed mesh structures on the membrane through FtsA anchoring along with expression of those proteins. Recording was started after 10 min incubation at 37 °C. Time indicates hh:mm:ss. Scale bar: 20 μm.

File name: Supplementary Movie 7

Description: Field view of FtsZ structures and Min waves within various sized lipid vesicles. Various FtsZ structures including FtsZ-ring were regulated by Min dynamic waves or static patterns through co-expression of all essential proteins, namely, FtsA, FtsZ-G55-Venus-Q56, mCherry-MinC, MinD, and MinE. Recording was started after 20 min incubation at 37 °C. Time indicates hh:mm:ss. Scale bar: 20 μm.

File name: Supplementary Movie 8

Description: Zoom-in view of a vesicle that formed FtsZ-ring driven by Min waves and constriction of the vesicle by FtsZ-ring. The vesicle is identical to the vesicle shown in the center-upper position in Supplementary Movie 7. (Left panel) 3D max projection of FtsZ-G55-Venus-Q56 and mCherry-MinC channel. Randomly distributed FtsZ mesh was then reorganized into ring structure by the pole-to-pole oscillation of Min waves. After formation of FtsZ-ring, Min waves stably kept pole-to-pole oscillation over 1 h, and FtsZ-ring constricted the vesicle. (Right panel) Equatorial plane of the vesicle visualized by Differential interference contrast. Images clearly show the “neck” of the constricted vesicle by FtsZ-ring. The vesicle itself slightly moves back and forth synchronically to the pole-to-pole oscillation of Min waves. Recording was started after 20 min incubation at 37 °C. Time indicates hh:mm:ss. Scale bar: 10 μm.

File name: Supplementary Movie 9

Description: 3D rotational view of a vesicle that formed FtsZ-ring driven by Min waves and constriction of the vesicle by FtsZ-ring. 3D max projected image was sequentially rotated along with Y-axis in 1 ° increment between each frame and 360 (0 ° – 359 °) frames were visualized as time-lapse. The image was captured started after 3 h of incubation at 37 °C. Scale bar: 5 μm.
