# Supplementary material for: In vitro assembly, positioning and contraction of a division ring in minimal cells
Source: Nat Commun. 2022 Oct 15;13:6098. doi: 10.1038/s41467-022-33679-x (PMC9569390; doi:10.1038/s41467-022-33679-x)
Supplement: Supplementary file 13 — Reporting Summary [file 41467_2022_33679_MOESM13_ESM.pdf]

nature portfolio

Corresponding author(s): Petra Schwille

Last updated by author(s): Sep 2, 2022

Reporting Summary

Nature Portfolio wishes to improve the reproducibility of the work that we publish. This form provides structure for consistency and transparency in reporting. For further information on Nature Portfolio policies, see our Editorial Policies and the Editorial Policy Checklist.

Statistics

For all statistical analyses, confirm that the following items are present in the figure legend, table legend, main text, or Methods section.

n/a	Confirmed	
☐	☒	The exact sample size (*n*) for each experimental group/condition, given as a discrete number and unit of measurement
☒	☐	A statement on whether measurements were taken from distinct samples or whether the same sample was measured repeatedly
☒	☐	The statistical test(s) used AND whether they are one- or two-sided<br>*Only common tests should be described solely by name; describe more complex techniques in the Methods section.*
☒	☐	A description of all covariates tested
☒	☐	A description of any assumptions or corrections, such as tests of normality and adjustment for multiple comparisons
☐	☒	A full description of the statistical parameters including central tendency (e.g. means) or other basic estimates (e.g. regression coefficient) AND variation (e.g. standard deviation) or associated estimates of uncertainty (e.g. confidence intervals)
☒	☐	For null hypothesis testing, the test statistic (e.g. *F*, *t*, *r*) with confidence intervals, effect sizes, degrees of freedom and *P* value noted<br>*Give P values as exact values whenever suitable.*
☒	☐	For Bayesian analysis, information on the choice of priors and Markov chain Monte Carlo settings
☒	☐	For hierarchical and complex designs, identification of the appropriate level for tests and full reporting of outcomes
☐	☒	Estimates of effect sizes (e.g. Cohen's *d*, Pearson's *r*), indicating how they were calculated

Our web collection on statistics for biologists contains articles on many of the points above.

Software and code

Policy information about availability of computer code

Data collection	Fiji (v1.53f) and imageJ custom macro code were used for all data collection and described in the text
Data analysis	Fiji (v1.53f) and imageJ custom macro code were used for all data analysis and described in the text

For manuscripts utilizing custom algorithms or software that are central to the research but not yet described in published literature, software must be made available to editors and reviewers. We strongly encourage code deposition in a community repository (e.g. GitHub). See the Nature Portfolio guidelines for submitting code & software for further information.

Data

Policy information about availability of data

All manuscripts must include a data availability statement. This statement should provide the following information, where applicable:

- Accession codes, unique identifiers, or web links for publicly available datasets
- A description of any restrictions on data availability
- For clinical datasets or third party data, please ensure that the statement adheres to our policy

All data sets for statistical graphs are provided in source data. Original image files are available upon reasonable request since most of them are quite large data (more than 10 GB volume) and therefore not suitable to deposit in public repository.

Field-specific reporting

Please select the one below that is the best fit for your research. If you are not sure, read the appropriate sections before making your selection.

☒ Life sciences ☐ Behavioural & social sciences ☐ Ecological, evolutionary & environmental sciences

For a reference copy of the document with all sections, see nature.com/documents/nr-reporting-summary-flat.pdf

Life sciences study design

All studies must disclose on these points even when the disclosure is negative.

Sample size	Sample sizes were not predetermined, however, were taken according to the standards of the field (e. g. n>100 lipid vesicles for classification of wave patterns or cell-free expression yield of sfGFP, from at least three independent biological replicates), which ensures sufficient statistics for characterization of molecular dynamics of studied protein systems.
Data exclusions	Criteria to exclude data was determined by the use of vesicles without lipid or protein aggregation and also only high quality data was used in our work.
Replication	Samples were taken at least three independent biological replicates on different days with similar results. Experiments belong to, at least, three independent samples. All the experiments were highly reproducible following the frequencies described in the text.
Randomization	Randomization was not applicable in this study since all experiments were performed and compared under well defined conditions with at least three independent repeats. Also, human or animal samples were not analyzed in the study and randomization is not often used in the related studies.
Blinding	Blinding was not applicable in this study since all experiments were performed and compared under well defined conditions with at least three independent repeats. Blinding in qualitative analyses (e. g. pattern classification) were not needed because the differences among different patterns were visually apparent and therefore were not required any subjective judgments. Other statistic analyses were only quantitative (e. g. aspect ratio of deformed vesicles, GTPase activity of FtsZ, etc.) and not applicable to blinding. Blinding is also not often used in the related studies

Reporting for specific materials, systems and methods

We require information from authors about some types of materials, experimental systems and methods used in many studies. Here, indicate whether each material, system or method listed is relevant to your study. If you are not sure if a list item applies to your research, read the appropriate section before selecting a response.

Materials & experimental systems

n/a	Involved in the study	
☒	☐	Antibodies
☒	☐	Eukaryotic cell lines
☒	☐	Palaeontology and archaeology
☒	☐	Animals and other organisms
☒	☐	Human research participants
☒	☐	Clinical data
☒	☐	Dual use research of concern

Methods

n/a	Involved in the study	
☒	☐	ChIP-seq
☒	☐	Flow cytometry
☒	☐	MRI-based neuroimaging
